# Knowledge and Practices of Community Pharmacists in Topical Dermatological Treatments

**DOI:** 10.3390/ijerph18062928

**Published:** 2021-03-12

**Authors:** Ana Teixeira, Maribel Teixeira, Maria Teresa Herdeiro, Viviana Vasconcelos, Rita Correia, Maria Fernanda Bahia, Isabel F. Almeida, Diogo Guedes Vidal, Hélder Fernando Pedrosa e Sousa, Maria Alzira Pimenta Dinis, Vera Almeida

**Affiliations:** 1 CESPU, Institute of Research and Advanced Training in Health Sciences and Technologies, Rua Central de Gandra 1317, 4585-116 Gandra, Portugal; ana.teixeira@iucs.cespu.pt (A.T.); viviana.vasconcelos@ics.ufpa.br (V.V.); a22562@alunos.cespu.pt (R.C.); vera.almeida@iucs.cespu.pt (V.A.); 2 UCIBIO/REQUIMTE, MedTech-Laboratory of Pharmaceutical Technology, Department of Drug Sciences, Faculty of Pharmacy, University of Porto, Rua de Jorge Viterbo Ferreira 228, 4050-313 Porto, Portugal; fgbahia@ff.up.pt; 3 Department of Medical Sciences—Institute of Biomedicine (iBiMED), University of Aveiro, Campus Universitario de Santiago, Agra do Crasto—Edificio 30, 3810-193 Aveiro, Portugal; teresaherdeiro@ua.pt; 4 UFP Energy, Environment and Health Research Unit (FP-ENAS), University Fernando Pessoa (UFP), Praça 9 de Abril 349, 4249-004 Porto, Portugal; diogovidal@ufp.edu.pt (D.G.V.); madinis@ufp.edu.pt (M.A.P.D.); 5 Department of Mathematics (DM. UTAD), University of Trás-os-Montes and Alto Douro, Quinta de Prados, 5001-801 Vila Real, Portugal; hfps@utad.pt

**Keywords:** community pharmacist, treatment adherence, disease management, pharmacists’ knowledge

## Abstract

The connection between pharmacists’ knowledge and practice on the provided information to patients about dermatoses and their treatment is insufficiently characterized. Furthermore, pharmacists’ contributions in counselling and in promoting adherence to topical treatment is not fully understood. This study has three main objectives. It aims to identify the knowledge and practices of pharmacists about dermatoses and their treatment, and to compare the perspective of pharmacists with that of patients regarding treatment information, with the future goal of establishing guidelines on the communication of dosage regimen instructions to dermatological patients and promotion of adherence to treatment, filling a gap. A cross-sectional, exploratory, and descriptive study was carried out. Based on experts’ prior knowledge and extensive collected literature information, two questionnaire protocols, one for pharmacists and another one for patients, were designed. Exploratory factor analysis (EFA) and confirmatory factor analysis (CFA) were carried out in relation to the pharmacists’ questionnaire for instrument validation. The results indicate that knowledge of pharmacists regarding dermatoses and their treatment is considered acceptable. Most of the pharmacists were reported to provide information to patients. Oppositely, patients reported not to have receive it. This is an important issue because pharmacists play a primary role in the management of several diseases. As non-adherence can be triggered by poor understanding of the dosing instructions, pharmacists’ communication practices play an important role in improving this hinderance. Results from this study identified pharmacist–patient communication gaps, so the development of guidelines to improve the transmission of clear dosage regimen instructions and knowledge about patient’s disease are of paramount importance. Training programs for continuous education of pharmacist should be implemented to solve the identified communication problems found in this study.

## 1. Introduction

Dermatoses are pathologies of high prevalence, with mental co-morbidity for which cutaneous medications are often a first-line therapeutic option [1]. The clinical effectiveness of these drugs is conditioned by treatment adherence [2]. In addition to the importance of adherence in the patient’s health, non-adherence has a high economic impact, due to the overuse of healthcare resources [3]. Adherence is an area of growing concern in the treatment of chronic diseases, particularly skin diseases [4] and the World Health Organization (WHO) has considered it a priority area of activity [5]. Some of the factors that affect treatment adherence are specific to topical medications, such as the mechanical properties of the formulations [6] and the difficulty in establishing clear dosage instructions [7]. The lack of knowledge about the dose to be applied was recognized as a factor that negatively influences treatment adherence [8,9].

Health professionals have an important role in promoting treatment adherence [10]. The pharmacist is the health professional who has the best skills to guide, educate and instruct the patient on the correct use of medicines, clarifying doubts and favouring adherence and the clinical success of the prescribed treatment [11,12]. Young et al. [13] found that pharmacists did not routinely direct patients to medicine information websites and thought leaflets might worry patients about possible side effects. Aimaurai et al. [14] proposed that community pharmacists could offer a Medicines Use Review service to ensure the quality use of medicines in the community after recognizing the unmet needs of patients for information on medicine.

The knowledge of community pharmacists about the characteristics of chronic dermatoses and their therapeutic regimens is unknown, which may hinder their role as players in plans to promote adherence to dermatological therapy. The transmission of health information is most effective when its contents are specifically targeted at a person or population group and when the message is well delimited, highlighting the benefits and costs associated with behaviours and decision making [15].

This study aims to identify the knowledge of pharmacists about dermatoses and their treatment, and to compare the perspectives of pharmacists with those of patients regarding treatment information, with the future goal of establishing clear guidelines on the communication of dosage regimen instructions by healthcare professionals to dermatological patients and promote treatment adherence.

## 2. Materials and Methods

### 2.1. Design and Procedures

This is a cross-sectional, exploratory, and descriptive study. Two questionnaire protocols were designed by the authors: one for the pharmacists and another one for the patients. For the generation of items included in the questionnaires, an expertise panel, comprised by 6 pharmacists, 3 psychologists and 1 dermatologist, was set, and the most prevalent dermatoses in Portugal were considered. The pool of items to include was decided, based on the panel professional experience and theoretical knowledge, also relying on an extensive systematic review of the literature and qualitative patients focus interviews. The counselling background is considered, based on the extensive experience of the panel. After pre-testing and validation, final versions of the questionnaires were then obtained. The participants, pharmacists and patients, covering the entire geographical mainland Portugal area, from the north to the south.

The patients and pharmacists’ protocols were applied in self-administered form through online procedures, in compliance with ethical standards and disclosed through the Portuguese Psoriasis Patient Association (PSO-Portugal) [16] and the Portuguese Pharmaceutical Society (Ordem dos Farmacêuticos—OF) [17], respectively, between 2018 and 2019. The eligibility criteria for patients included to be 18 years of age or older; to have a clinical diagnosis of psoriasis; to belong to PSO-Portugal and/or to the OF. The eligibility criteria for pharmacists were to be a pharmacist, recognized by the OF [17], and work in community pharmacies. All procedures were in accordance with the ethical standards of the Ethics Committee of *Instituto Universitário de Ciências da Saúde* (IUCS/CESPU) [18], Portugal, no specific reference assigned, date acting as reference identification (17 March 2017), approved and made available online, and with the 1964 Helsinki declaration and its later amendments or comparable ethical standards. The study was also approved by the Portuguese Data Protection Authority (Comissão Nacional de Proteção de Dados—CNPD) [19], Portugal, date acting as reference identification (26 September 2017). Informed consent was made available and obtained from all individual participants included in the study.

### 2.2. Participants

#### 2.2.1. Pharmacists

The sample consists of 149 pharmacists working in community pharmacy, mostly female (83.2%), with a mean age of 37.99 years (*SD* = 9.90; *Min* 24–*Max* 72) and with a mean of education graduation of 12.89 years (*SD* = 9.45; *Min* 1–*Max* 28).

#### 2.2.2. Patients

The sample of patients is composed of 44 participants, of which 67.4% are female. The mean age is 50.65 years old (*SD* = 16.075; *Min* 9–*Max* 76); 47.7% of the sample has an academic degree and 31.8% has only secondary education.

### 2.3. Instruments

The pharmacists’ protocol included a sociodemographic questionnaire, assessing age, gender and years from graduation and another questionnaire, the Dermatologic Topical Treatment Knowledge (DTTK) to assess the pharmacists’ knowledge regarding dermatoses and their treatment and treatment adherence (Table 1). The first ten questions in this questionnaire aimed to assess the knowledge of pharmacists in relation to dermatoses and their treatment (subscale 1) and the remaining questions assessed pharmacists’ knowledge in relation to dosage regimen instructions and treatment adherence (subscale 2). It was also possible to obtain a total score value. All questions were designed in such a way that each question corresponded to several answering options, which were assigned a value as it approached more or distanced itself from the right answer that was indicative of knowledge. The higher the score, the greater the knowledge of each pharmacist in relation to the topic under consideration.

The questionnaire started asking about the most prevalent chronic dermatoses in Portugal. The second question addresses the factors related to prevalence of chronic dermatoses. A group of 4 questions assesses the most characteristic lesions of psoriasis, atopic dermatitis, seborrheic dermatitis and acne. Another group of 4 questions measures the knowledge about the most prescribed pharmacotherapeutic groups for the treatment of psoriasis, atopic dermatitis, seborrheic dermatitis and acne. A question measures the information about the factors that influence adherence to skin treatment of chronic dermatoses. Another question assesses the most appropriate instructions to explain the dose of topical medicine. A group of 3 questions included the assessment of the behaviour to instruct the patient about the duration of corticosteroid, immunomodulators and anti-infectious topical treatment. The next 2 questions rely on asking the pharmacist if they instruct the patient on the mode and the frequency of application of topical medicines. The two followed questions address the indication of clear and precise dosage regimen instructions for topical treatment and the factors that influence it. The last 2 questions are related to the prevalence of adherence to topical treatment of chronic dermatoses and the perception of the importance of pharmaceutical intervention in the improvement of the disease. The patients’ protocol also included a sociodemographic questionnaire, assessing gender, age and education and a questionnaire regarding the interaction with pharmacists.

### 2.4. Data Analyses

The data were analysed using the Statistical Program for Social Sciences SPSS IBM, version 25 [20]. Descriptive statistics, including frequency, percentage, mean, standard deviation, was used to characterize the sample and the pharmacists’ and patients’ responses. Kurtosis and skewness values were calculated to assess the normality distribution of the sample. Cronbach’s alpha was calculated to assess the instrument’s reliability. Exploratory factor analysis (EFA) and confirmatory factor analysis (CFA) were carried out in relation to the pharmacists’ questionnaire for instrument validation.

## 3. Results

### 3.1. Pharmacists

Table 1 presents the descriptive values of the pharmacists’ responses, as well as the Cronbach’s alpha value for all items and for the two subscales of the questionnaire. The skewness and kurtosis values are below the limits established by Kline [21], respectively 3 and 10, suggesting the normal distribution of responses to the items. The value of Cronbach’s alpha for the total of the questionnaire is at the limit of the acceptable (0.70) [22] as well as the alpha of the dosage regimen instructions and adherence subscale. According to Hair et al. [22], the dermatoses and treatments subscale has an unacceptable Cronbach’s alpha value (Table 1).

Regarding the pharmacists’ answers, the three most prevalent chronic dermatoses in Portugal were atopic dermatitis, psoriasis and seborrheic dermatitis, and the prevalence of chronic dermatoses varies mainly with genetic factors, environmental factors, age and lifestyle. The most characteristic lesions of psoriasis were desquamative papules/plaques followed by erythema. Of atopic dermatitis are the erythema and desquamative papules/plaques, of seborrheic dermatitis are the desquamative papules/plaques and erythema, and of acne are the comedones and pustules. Participants revealed that corticosteroids are the most prescribed pharmacotherapeutic group for the treatment of psoriasis, atopic dermatitis and seborrheic dermatitis. Antibacterials are the most prescribed pharmacotherapeutic group for the treatment of acne. Participants recognized that the severity of the disease and the patient’s socioeconomic conditions are the factors that most influence the treatment adherence, an important outcome. They referred that *apply in thin layer* was the most appropriate instruction. Most pharmacists always instruct the patient about the duration of corticosteroid skin treatment and its associations, of skin treatment with immunomodulators and of anti-infectious skin treatment. Half of the sample report to always instruct the patient on how to apply the medicine and on how often to apply topical medicines. Almost all participants considered that the indication of clear and precise dosing instructions for dermatological skin treatments increases the effectiveness of skin drug treatment, increasing the treatment adherence with topical medicines and allowing to minimize the adverse effects of skin medications. Most of the participants considered that the indication of dosing instructions depends on the type of treatment. Participants thought adherence to topical treatment of chronic dermatoses is mostly between 50 and 69%. One third of the sample stated that adherence to topical treatment of chronic dermatoses can be increased with pharmaceutical intervention (Table 2).

The answers considered correct were scored with 2 points and the incorrect answers with zero. The acceptable responses were scored with 1 point, thus allowing to establish a score that assesses the knowledge of pharmacists. Acceptable knowledge has been found, i.e., the total score was higher than 50% of maximum value (e.g., total results vary from 0 to 201 and thus 100.5 score was considered as acceptable). The obtained results show that pharmacists have more than acceptable knowledge regarding dermatoses and their treatment and dosage regimen instructions and treatment adherence (145.59 corresponding to 72.4%).

The factorability of the 21 pharmacists’ questionnaire items was examined. The Kaiser–Meyer–Olkin [23] measure of sampling adequacy was 0.80 (above the recommended value of 0.6), and Bartlett’s test of sphericity [24] was significant (*χ*^2^ (210) = 1217.92, *p* < 0.000). Finally, the communalities were all above 0.40, confirming that each item shared some common variance with other items. Thus, EFA was considered to be suitable with all 21 items (Table 3).

Principal components analysis, with varimax rotation, was used with prior determination of two factors. Eigenvalues indicated that the first two factors explained 22 and 8% of the variance, respectively. A total of four items were removed because they did not contribute to a simple factor structure and failed to meet a minimum criterion of having a primary factor loading of 0.4 or above, and no cross-loading of 0.3 or above (items 1, 2, 11 and 12). A new analysis of principal components was carried out, now with 17 items and prior determination of two factors. The Kaiser–Meyer–Olkin measure of sampling adequacy was 0.82 and Bartlett’s test of sphericity was significant (*χ*^2^ (120) = 1089.80, *p* < 0.000). Eigenvalues indicated that the first two factors explained 26 and 10% of the variance, respectively. However, items 9 and 20 were removed because they did not contribute to a simple factor structure and failed to meet a minimum criterion of having a primary factor loading of 0.4 or above. The last analysis of principal components was carried out, now with 15 items and prior determination of two factors. The Kaiser–Meyer–Olkin measure of sampling adequacy was 0.83 and Bartlett’s test of sphericity was significant (*χ*^2^ (105) = 1051.26, *p* < 0.000). Eigenvalues indicated that the first two factors explained 29 and 11% of the variance, respectively, with a total of 40% (Table 4). Cronbach’s alpha values rose slightly with pharmacist’s knowledge and adherence subscale, obtaining 0.77, and dermatoses and treatment subscale, obtaining 0.44 and total 0.65.

**Figure 1:**
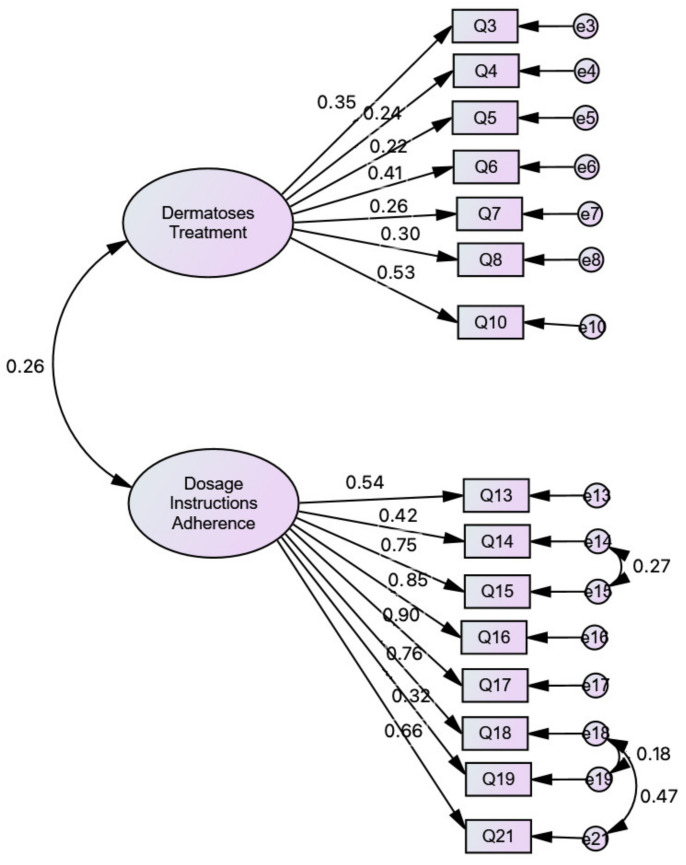
Model Fit of the Pharmacists’ Knowledge Questionnaire in a sample of pharmacists whose workplace is the community pharmacy: *χ^2^* = 154.008; *df* = 86; χ^2^/*df* = 1.791; CFI = 0.930; TLI = 0.915; RMSEA = 0.059; PCLOSE = 0.163.

CFA was used to test whether the pharmacists’ questionnaire measures are consistent with the researchers’ understanding of the nature of that construct. As presented in Figure 1, the model shows a good fit. According to Marôco [25], the sample size is within the required parameters (*n* = 200–400) regarding the Maximum Likelihood (ML) method in Structural Equation Modelling (SEM). There is no missing data. To verify the existence of outliers, the Mahalanobis squared distance [26] (*p1* and *p2* < 0.001) was used [21]. To test the items’ multicollinearity, Spearman coefficients were calculated, according to a reference value of 0.80 [27]. To assess the goodness-of-fit of the model to the global correlation frame, Comparative Fit Index (CFI) and Tucker–Lewis index (TLI), Parsimonious Fit Indices representing adjustments, values greater than 0.9 are indicative of a good fit. Values of *χ^2^*/df = ~2 and the Root Mean Square Error of Approximation (RMSEA) [28] < 0.08 were considered, indicating a good model fit. The refinement of the questionnaire original model was performed from the values of the Modification Indices (MI), for the Lagrange multipliers (LM) [29], considering that trajectories and/or correlations with LM > 11 (*p* < 0.001) indicate significant variation in the quality of the model [25].

### 3.2. Patients

Most of the sample of patients (77.8%) is undergoing treatment for psoriasis and the remaining participants are being treated for acne, rosacea and seborrheic dermatitis. Most (79.5%) are undergoing treatment directed by a dermatologist. The others do so, advised by their General Physician and/or pharmacist. A significant part of the sample (36.4%) was consulted and medicated in a private physician’s office, and another part (34.1%) in a public hospital, while the rest were consulted in private hospitals and health centres. Only 25.6% of the sample is undergoing the dermatological treatment for the first time, and 74.4% of the sample is undergoing continued treatment.

Only 11.4% of the sample reported receiving oral information at the pharmacy about the dose of the medication to use. In total, 18.2% reported to have received information at the pharmacy about the duration of treatment and the number of times needed to apply the medicine; 15.9% reported having had information at the pharmacy about the mode of application of the medicine.

## 4. Discussion

According to the results obtained, regarding pharmacists, atopic dermatitis has been identified as the most prevalent chronic dermatosis in Portugal, in line with the Oliveira and Torres [30] study, erythema being its most frequent clinical manifestation, as stated by Siegfried and Hebert [31]. Corticosteroids are reported as the most prescribed drugs for this disease, corroborating the Pona et al. [32] study. Psoriasis is the second most dermatosis prevalent, the most frequent lesions being desquamative papules/plaques; Corticosteroids are also the most prescribed drugs for psoriasis treatment, which is in accordance with Dolz-Pérez and colleagues [33] study. In this study, seborrheic dermatitis and acne were identified, respectively, as the third and fourth most common dermatoses in Portugal.

Desquamative papules/plaques and erythema have been pointed out as the most frequent characteristics of seborrheic dermatitis, and comedones considered to be the most frequent characteristic of acne, in line with Borda, Perper and Keri [34] study. Again, corticosteroids are the most prescribed drugs for seborrheic dermatitis, and antibacterials the most prescribed drugs for acne, corroborating Borda and colleagues [34] and Brown [35] studies, respectively.

Genetic factors are primarily responsible for the prevalence of chronic dermatoses [36]. The participants in this study recognized that the severity of the disease and the patient’s socioeconomic conditions are the factors that most influence the treatment adherence in accordance with reported by Eicher et al. [37]. Pharmaceutics believe that the most appropriate instructions to explain to the patient the dose of topical medicine to be applied is *apply in thin layer*, corroborating Goman findings [38]. Most of the participants (>67.4%) stated that they always/almost always instruct the patient about (a) the duration of corticosteroid skin treatment and its associations; (b) the duration of anti-infectious skin treatment; (c) how to apply the medicine; (d) how often to apply cutaneous medicines, in accordance with Tucker and Stewart [39] results. The participants considered that the indication of clear and precise dosage regimen instructions for dermatological skin treatments are important because contributes to increase adherence and effectiveness of topical treatment and to minimize the adverse effects of topical medicines, in line with Yamaura [40] observations. According to pharmacists, the indication of dosage regimen instructions, i.e., frequency, duration and dose of the medicinal product to be administered, mainly depends on the type of treatment, e.g., corticotherapy, antibiotic therapy, as Teixeira and colleagues [41] also stated. Although pharmacist knowledge is acceptable, the results obtained highlight the need to promote the communication of this information to the patients, aiming to improve adherence and clinical outcome of treatment. For this purpose, training programs and guidelines adapted to pharmacists’ needs should be developed and implemented as part of continuous education of these health professionals.

The knowledge of pharmacists regarding dermatoses and their treatment is considered acceptable. This is an important subject because community pharmacists play a primary role in the management of several diseases [42,43] since, these health professionals, are easily accessible and have knowledge to clarify dosing instructions and the opportunity to emphasize the importance of treatment adherence in compliance with the therapeutic regime for the effectiveness of treatment. Different studies demonstrate the relevance of pharmacist interventions in the management of different dermatoses. Aishwarya et al. [44] reported that medication adherence among psoriasis patients was improved after pharmacist education and counselling intervention. Tucker and Stewart et al. [39] stated the enhancement of patients’ psoriasis knowledge, minimizing the severity of the disease and bettering quality of life, following education intervention delivered by community pharmacist.

Regarding patients, only a quarter of the sample reported receiving oral information at the pharmacy about the dose of medication to use. Almost a third of the sample have received information at the pharmacy about the duration of treatment and the number of times to apply the medicine and less than a third reported having had information at the pharmacy about the mode of its application.

Discrepancies were found between what pharmacists claim to report to patients and what patients claim to have been reported by pharmacists, which is in accordance with our previous study, since most patients announced that they did not receive the reinforcement of the dosing instructions when they fill the prescriptions at the pharmacy [45].

As Tulsky and colleagues [46] stated, poor communication by healthcare professionals contributes to physical and psychological suffering in patients. Despite this, pharmacists reported instructing patients regarding topical dosage regimens and hold acceptable knowledge of this issue, which is in line with, Johnson, Moser, and Garwood [47], who stated that the majority of patients reported that they did not receive that instructions, suggesting the need to outline strategies to improve communication between pharmacist and patients, in order to promote adherence and clinical outcome of the treatment.

## 5. Conclusions

It is recognized that the link between the knowledge and practice of pharmacists in what concerns the information given to patients about dermatoses and corresponding treatment is not fully characterized. It is also recognized that the pharmacists’ contribution in counselling and in adherence promotion to topical treatment is not fully understood. Three main objectives were then considered: identification of the knowledge of pharmacists about dermatoses and their treatment; comparison of the perspective of pharmacists with that of patients regarding treatment information; future goal of establishing clear guidelines on the communication of dosage regimen instructions by healthcare professionals to dermatological patients and promote treatment adherence.

An expertise panel, including pharmacists, psychologists and dermatologists was set. Based on their expertise and extensive literature review, two questionnaire protocols, orientated to pharmacists and patients, were designed, after initial pre-testing and validation. EFA and CFA were applied to the results.

The knowledge of pharmacists regarding dermatoses and their treatment is considered acceptable, which is an important result as pharmaceuticals play a primary role in the management of several diseases. Discrepancies were found regarding the communication of instructions by pharmacists, since most pharmacists reported to provide information to patients but only a low percentage of patients reported to have received it. Concerning practical implications, most of the skin diseases can be effectively treated with topical medicines. However, non-adherence to topical treatment, caused by poor understanding of the dosing instructions, leads to clinical ineffectiveness.

This study thus assumes particular importance because it allows one to identify communication gaps between pharmacists and patients, with negative implications in the adherence and clinical outcome of topical treatments. Future studies focused on the establishment of guidelines to improve the communication of dosage regimen instructions by pharmacists to dermatological patients, could overcome the problems recognized in this study. Furthermore, training programs for the continuous education of the pharmacist should be implemented to solve the identified problems found in this study.

## Figures and Tables

**Table 1:** Pharmacists’ answers, total and subscales description (*N* = 230).

Questions	Theoretical *Min*–*Max*	*Min*	*Max*	*M*	*SD*	*S_kw_*	*K_rt_*	*α*
**Q1** Prevalent chronic dermatoses	0–13	4	10	8.50	1.81	−1.15	−0.25	
**Q2** Factors of chronic dermatoses	0–14	2	14	10.30	2.63	−0.24	−0.04	
**Q3** Typical psoriasis lesions	0–13	8	12	11.07	1.53	−1.37	0.14	
**Q4** Typical atopic dermatitis lesions	0–13	7	12	9.77	1.89	0.15	−1.88	
**Q5** Typical seborrheic dermatitis lesions	0–14	6	14	11.97	2.45	−0.79	−0.38	
**Q6** Typical acne lesions	0–12	2	11	9.22	1.57	−1.84	2.28	
**Q7** Prescribed medicines for psoriasis	0–14	7	12	10.96	1.24	−1.72	2.16	
**Q8** Prescribed medicines for atopic dermatitis	0–14	2	10	8.37	1.54	−1.04	0.14	
**Q9** Prescribed medicines for seborrheic dermatitis	0–15	4	12	10.36	1.83	−1.25	0.92	
**Q10** Prescribed medicines for acne	0–16	4	12	11.34	1.50	−2.12	3.41	
**Q11** Factors adherence chronic dermatoses	0–12	1	10	7.42	1.63	−1.77	2.54	
**Q12** Appropriate instructions of medicines	0–9	0	8	5.37	1.84	−0.35	−0.50	
**Q13** Instruction duration corticosteroids	0–2	0	2	1.81	0.50	−2.63	5.99	
**Q14** Instruction duration immunomodulators	0–2	0	2	1.19	0.91	−0.39	−1.67	
**Q15** Instruction duration anti-infectious	0–2	0	2	1.59	0.72	−1.43	0.43	
**Q16** How to apply the medicine	0–2	0	2	1.63	0.72	−1.60	0.84	
**Q17** How often apply cutaneous medicines	0–2	0	2	1.58	0.76	−1.43	0.26	
**Q18** Indications of clear and precise dosing	0–10	4	10	8.97	2.22	−1.75	1.14	
**Q19** Dosing instructions depend on...	0–10	3	9	5.98	1.97	−0.23	−1.68	
**Q20** Dimension of adherence to topical treatment	0–10	4	8	6.79	1.67	−0.85	−1.02	
**Q21** Increasing adherence to topical treatment	0–2	0	2	1.41	0.75	−0.85	−0.74	
**Total**	0–201	96	168	145.59	12.05	−1.31	2.85	0.65
Dermatoses & treatment	0–138	74	115	101.85	7.36	−0.96	1.50	0.41
Dosage instructions & adherence	0–63	21	55	43.74	7.77	−1.24	1.06	0.72

*Min* = Minimum; *Max* = Maximum; *M =* Mean; *SD* = Standard Deviation; *S_kw_* = Skewness; *K_rt_* = Kurtosis; *α = Cronbach Alfa*.

**Table 2:** Distribution of pharmacists’ answers.

Questions	Pharmacists’ Answers	%
**Q1** In your opinion, which are **the three most prevalent chronic dermatoses in Portugal**?	**Dermatitis**	**84.3**
	Psoriasis	67.0
	Seborrheic dermatitis	58.7
	Acne	56.5
	Androgenic Alopecia	8.7
	Scabies	2.6
**Q2** In your opinion, the **prevalence of chronic dermatoses varies mainly with** (tick the three answers you consider most relevant):	**Genetic factors**	**87.4**
	Environmental factors	65.2
	Age	59.1
	Lifestyle	53.9
	Gender	17.4
	Socioeconomic factors	13.9
	Educational level	1.3
**Q3** In your opinion, the **most characteristic lesions of psoriasis** are (tick the two answers you consider most relevant):	**Desquamative papules/plaques**	**97.4**
	Erythema	67.4
	Pustules	15.2
	Hyperpigmentation	11.3
	Ulcers	6.1
	Blisters	1.3
	Comedones	0.4
**Q4** In your opinion, the **most characteristic lesions of atopic dermatitis** are (tick the two answers you consider most relevant):	**Erythema**	**94.8**
	Desquamative papules/plaques	77.0
	Pustules	20.0
	Ulcers	15.2
	Comedones	12.6
	Hyperpigmentation	6.1
	Blisters	6.1
**Q5** In your opinion, the **most characteristic lesions of seborrheic dermatitis** are (tick the two answers you consider most relevant):	**Desquamative papules/plaques**	**77.0**
	Erythema	70.4
	Pustules	20.0
	Comedones	12.6
	Hyperpigmentation	6.1
	Blisters	6.1
	Ulcers	3.9
**Q6** In your opinion, the **most characteristic lesions of acne** are (tick the two answers you consider most relevant):	**Comedones**	**95.2**
	Pustules	77.4
	Blisters	8.3
	Erythema	5.7
	Ulcers	4.8
	Hyperpigmentation	3.0
	Desquamative papules/plaques	3.0
**Q7** In your opinion, what are the **most prescribed pharmacotherapeutic** groups for the treatment of **psoriasis** (mark two answers you consider most relevant):	**Corticosteroids**	**86.5**
	Keratolytics	43.5
	Vitamin D analogues	32.6
	Immunomodulators	23.5
	Retinoids	7.8
	Antibacterials	1.7
	Antifungals	0.4
**Q8** In your opinion, what are the **most prescribed pharmacotherapeutic** groups for the treatment of **atopic dermatitis** (marked two answers you consider most relevant):	**Corticosteroids**	**95.2**
	Immunomodulators	35.2
	Antibacterials	20.0
	Keratolytics	13.9
	Retinoids	10.9
	Antifungals	9,6
	Vitamin D analogues	8.3
	Antivirals	0.4
**Q9** In your opinion, what are the **most prescribed pharmacotherapeutic** groups for the treatment of **seborrheic dermatitis** (marked two answers you consider most relevant):	**Corticosteroids**	**63.9**
	Antifungals	53.5
	Keratolytics	47.8
	Antibacterials	13.5
	Retinoids	7.0
	Immunomodulators	4.3
	Vitamin D analogues	2.6
**Q10** In your opinion, what are the **most prescribed pharmacotherapeutic** groups for the treatment of **acne** (marked two answers you consider most relevant):	**Antibacterials**	**86.1**
	Retinoids	72.2
	Keratolytics	19.6
	Corticosteroids	7.4
	Antifungals	3.0
	Vitamin d analogues	3.0
	Immunomodulators	1.7
**Q11** In your opinion, the **factors that influence adherence** to skin treatment of chronic dermatoses are (tick the three answers you consider most relevant)”	**Severity of the disease**	**84.8**
	**Socioeconomic conditions**	**84.3**
	Vehicle/base of the medicine	45.2
	The season	30.4
	The knowledge of dosage	25.7
	The skin type	9.1
	The medicine strength	5.7
**Q12** In accordance with your professional practice, refer to the **most appropriate instructions to explain to the patient the dose of topical medicine** to be applied (tick the two answers you consider most relevant)”	**Apply in thin layer**	**85.2**
	Apply an amount equivalent to the “fingertip unit” to an area approximately one palm	46.1
	Apply a pea-sized amount to each lesion	40
	Apply generously to the area to be treated	8.7
	Apply an amount equivalent to the “fingertip unit” to an approximate area of two palms	8.7
**Q13** According to your professional practice, do you usually **instruct the patient about the duration of corticosteroid skin treatment** and its associations?	**Always**	**63.9**
	Almost always	16.5
	Several times	6.1
	Sometimes	3.5
	Rarely	0.4
	Never	0.4
**Q14** According to your professional practice, do you usually **instruct the patient about the duration** of skin treatment with **immunomodulators**?	**Always**	**35.7**
	Almost always	21.7
	Several times	8.7
	Sometimes	6.5
	Rarely	15.7
	Never	8.3
**Q15** According to your professional practice, do you usually **instruct the patient about the duration** of **anti-infectious** skin treatment?	**Always**	**53.4**
	Almost always	18.3
	Several times	9.1
	Sometimes	4.3
	Rarely	2.2
	Never	1.7
**Q16** According to your professional practice, do you usually **instruct the patient on how to apply** the medicine?	**Always**	**50.0**
	Almost always	27.0
	Several times	6.1
	Sometimes	2.6
	Rarely	0.9
	Never	2.2
**Q17** According to your professional practice, do you usually **instruct the patient about how often to apply** cutaneous medicines?	**Always**	**53.5**
	Almost always	21.7
	Several times	5.2
	Sometimes	2.6
	Rarely	0.9
	Never	1.7
**Q18** In your opinion, **the indication of clear and precise dosing instructions** for dermatological skin treatments (please tick the three answers you consider most relevant) is:	**Increases the effectiveness of skin drug treatment**	**84.3**
	Contributes to increased treatment adherence with topical medicines	83.5
	Allows to minimize the adverse effects of skin medications	82.6
	Systemic treatments as these (skin application) are very safe	0.9
	Systemic treatments as these (skin application) are ineffective	0.9
**Q19** In your opinion, **the indication of dosing instructions** (frequency, duration and dose of the medicinal product to be administered) **depends on** (tick the two answers you consider most relevant):	**The participants choose of the type of treatment**	**62.6**
	The type of dermatosis	44.8
	The treatment complexity	21.3
	When the medicine is first used	17.0
	The existence of several affected anatomical zones	14.8
	The type of base/vehicle	9.6
**Q20** In your opinion, **adherence to topical treatment** of chronic dermatoses is:	Greater than 90%	1.7
	Between 70–90%	17.4
	**Between 50–69%**	**43.5**
	Between 30–49%	18.7
	Less than 30%	3.5
**Q21** According to your professional practice, **can adherence to topical treatment of chronic dermatoses be increased with pharmaceutical intervention?**	**Always**	**31.3**
	Almost always	26.1
	Several times	20.4
	Sometimes	6.1
	Rarely	0.9

**Table 3:** Exploratory factorial analysis (EFA).

Questions	Communalities	Component Matrix	
**Q1** Prevalent chronic dermatoses	0.640	0.182	0.113
**Q2** Factors of chronic dermatoses	0.481	0.225	0.150
**Q3** Typical psoriasis lesions	0.479	0.080	0.412
**Q4** Typical atopic dermatitis lesions	0.594	−0.063	0.394
**Q5** Typical seborrheic dermatitis lesions	0.635	−0.039	0.442
**Q6** Typical acne lesions	0.412	−0.044	0.558
**Q7** Prescribed medicines for psoriasis	0.653	0.169	0.342
**Q8** Prescribed medicines for atopic dermatitis	0.625	−0.041	0.411
**Q9** Prescribed medicines for seborrheic dermatitis	0.654	0.093	0.306
**Q10** Prescribed medicines for acne	0.506	0.168	0.576
**Q11** Factors adherence chronic dermatoses	0.407	0.341	0.375
**Q12** Appropriate instructions of medicines	0.494	0.089	0.218
**Q13** Instruction duration corticosteroids	0.652	0.610	0.313
**Q14** Instruction duration immunomodulators	0.553	0.517	0.056
**Q15** Instruction duration anti-infectious	0.732	0.758	0.169
**Q16** How to apply the medicine	0.723	0.831	0.058
**Q17** How often apply cutaneous medicines	0.810	0.869	−0.021
**Q18** Indications of clear and precise dosing	0.790	0.833	−0.015
**Q19** Dosing instructions depend on…	0.452	0.438	−0.065
**Q20** Dimension of adherence to topical treatment	0.545	0.230	0.083
**Q21** Increasing adherence to topical treatment	0.697	0.767	−0.091

**Table 4:** Final exploratory factorial analysis (EFA).

Questions	Component Matrix	
**Q3** Typical psoriasis lesions	0.081	0.447
**Q4** Typical atopic dermatitis lesions	−0.05	0.398
**Q5** Typical seborrheic dermatitis lesions	−0.03	0.369
**Q6** Typical acne lesions	−0.032	0.611
**Q7** Prescribed medicines for psoriasis	0.174	0.355
**Q8** Prescribed medicines for atopic dermatitis	−0.047	0.471
**Q10** Prescribed medicines for acne	0.179	0.635
**Q13** Instruction duration corticosteroids	0.612	0.274
**Q14** Instruction duration immunomodulators	0.529	0.053
**Q15** Instruction duration anti-infectious	0.775	0.210
**Q16** How to apply the medicine	0.834	0.084
**Q17** How often apply cutaneous medicines	0.872	0.015
**Q18** Indications of clear and precise dosing	0.829	−0.035
**Q19** Dosing instructions depend on…	0.437	−0.086

## Data Availability

As part of consenting to the study, survey respondents were assured that raw data would remain confidential and would not be shared. Descriptive data may be available upon request from the corresponding authors.
